# Study of single-level lumbar degenerative diseases treated by unilateral wiltse access with unilateral nail rod fixation assisted by a new automatic retraction device

**DOI:** 10.1186/s13018-022-03474-x

**Published:** 2023-01-27

**Authors:** Yapeng Sun, Wei Zhang, Fei Zhang, Jiaqi Li, Lei Guo

**Affiliations:** Department of Spinal Surgery, The Third Hospital of Hebei Medical University, No. 139 Ziqiang Road, Qiaoxi District, Shijiazhuang, 050000 Hebei China

**Keywords:** Wiltse approach, Unilateral fixation, Kidney-like fusion cage, TLIF, Lumbar degenerative diseases

## Abstract

**Objective:**

To evaluate the clinical efficacy of unilateral wiltse transforaminal lumbar interbody fusion (TLIF) combined with unilateral nail bar system fixation for single-level lumbar degenerative diseases with the assistance of a new automatic retraction device in a retrospective comparative study.

**Methods:**

A total of 46 patients with single-level lumbar degenerative diseases from September 2019 to December 2021 were retrospectively analyzed. Bilateral nail bar fixation with bullet-type fusion cage (ctrl group, 24 patients) and unilateral nail bar fixation on the affected side with kidney-like fusion cage (study group, 22 patients) were performed in TLIF via wiltse intermuscular approach assisted by a new automatic retraction device. The differences in intraoperative blood loss, operative time, intraoperative fluoroscopy time, postoperative drainage, bed rest, VAS score, ODI score, JOA score, serological creatine kinase (CK), the proportion of multifidus atrophy, modified Pfirrmann classification and intervertebral space height of the upper intervertebral disc were compared between the two groups based on clinical and imaging data.

**Results:**

Intraoperative bleeding, operative time, and postoperative drainage were significantly lower in study group than ctrl group, and there were no significant differences in bed rest time and intraoperative fluoroscopy time between them. In addition, there was no statistical difference in CK between the study group and the ctrl group at 24 and 48 h postoperatively. Moreover, no statistically significant difference was found in VAS score of low back pain, VAS score of lower limb pain, ODI index, modified Pfirrmann classification of the upper intervertebral disc and intervertebral space height of the upper intervertebral disc between two groups. The atrophy ratio of multifidus muscle was significantly lower in the study group.

**Conclusion:**

The new automatic retraction device assisted unilateral TLIF surgery with wiltse approach combined with unilateral nail bar fixation is a simple, effective and easy to master surgical method for single-level lumbar degenerative diseases.

## Introduction

Lumbar degenerative disc disease is a disease that sometimes causes low back pain or radiating pain from damaged discs in the spine [1]. Imaging manifestations of degenerative disc disease include narrowing of the disc space, loss of T2 signal intensity in the disc space, ligamentous changes, bone marrow changes, herniation, osteophyte formation, subluxation, and stenosis [2]. At present, the main treatment methods for degenerative lumbar disc disease are conservative treatment and surgery. However, exposing the degenerative lumbar segments during surgery is not easy.

In 1968, Wiltse pioneered the use of the muscle gap between the multifidus and longest muscles to expose the lumbar spine for interbody fusion of the lumbosacral segment, later referred to as the Wiltse approach [3, 4]. This gap not only allowed satisfactory exposure of the posterior lateral lumbar structures such as the transverse processes, articular joints and foramina, but also preserved the integrity of the interspinous and supraspinous ligaments [5]. Since then the Wiltse approach has been widely used in lumbar spine surgery. However, the lumbar spinal canal is difficult to expose due to the obstruction of the multifidus muscle and therefore decompression through this gap is difficult [6]. In order to reduce the muscle damage caused by the wiltse interbody approach, we designed a new automatic retraction device combined with unilateral nail bar fixation and a kidney-like fusion cage for the treatment of single-level lumbar degenerative diseases to investigate whether this method can effectively treat lumbar degenerative diseases and reduce muscle damage.

## Materials and methods

### Inclusion and exclusion criteria

Inclusion criteria: (1) patients were diagnosed with lumbar degenerative diseases, including lumbar disc herniation together with intervertebral instability, lumbar spinal stenosis, or lumbar spondylolisthesis; (2) patients had unilateral or bilateral lower limb symptoms (intermittent claudication or sciatica), and the symptoms were not relieved after 3 months of conservative treatment; (3) physical examination and imaging of the patient were in accordance with single-segment lumbar spine disease.

Exclusion criteria: Patients who have the following conditions (1) lumbar spondylolisthesis ≥ 2 degrees or with significant isthmic fissure; (2) experienced in lumbar spine surgery; (3) congenital developmental abnormalities of the lumbar spine; (4) infection, tumour or fracture of the diseased segment; (5) combination of other cardiovascular and cerebrovascular diseases that cannot tolerate surgery.

### Group setting

A total of 46 cases were included in the study group and were treated by Wiltse approach surgery. 22 cases were treated by unilateral nail rod fixation combined with kidney-like peek fusion placement on the affected side (study group) and 24 cases were treated by bilateral nail rod system fusion combined with conventional bullet fusion cage (ctrl group). Cases with protrusion or stenosis in the central region were included in the ctrl group, and cases with protrusion or stenosis in the lateral saphenous fossa, intervertebral foramen and extradural region were included in the study group.

### Surgical method

Study group: The operation was performed under general anaesthesia in the prone position. The C-arm X-ray machine is used for fluoroscopic positioning to identify the target segment and a posterior midline skin incision of approximately 7 cm is made. The skin and subcutaneous tissues were incised in sequence, and the subcutaneous layer and the lumbodorsal fascia were moderately freed along the subcutaneous layer, and the lumbodorsal fascia was incised longitudinally at 2.5–3 cm from the posterior midline. A finger probe is used to bluntly separate the muscular space between the multifidus and longest muscles and to access the articular eminence. A hemi-plate puller is used to assist in exposing the surgical area and inserting the pedicle screw under fluoroscopy. New automatic retractor is used to widen the muscle gap and an electroknife is used to subperiosteally separate the multifidus muscle attached to the vertebral plate to the base of the spinous process, resetting the pull hook to fully expose the surgical field. The laminar forceps are used to bite away part of the affected lamina, the inferior and superior articular processes and the apical and medial parts of the superior articular processes, and to remove the affected ligamentum flavum. The nerve stripper is used to separate the adhesions, the nerve roots are protected with cotton pads, the diseased disc is removed and the cartilage endplates are scraped. The intervertebral space is flushed and bone grafted and a kidney-like intervertebral fusion device filled with allograft bone and bone morphogenetic protein is placed. Kidney-like intervertebral fusion device placed horizontally in the anterior middle of the vertebral body. Then the automatic retractor is removed, the connecting rod is attached and the nail cap is locked with moderate pressure. The drainage tube was placed and then tightly sutured layer by layer.

Ctrl group: posterior nail placement with bilateral staple fixation after access to the muscular space on the healthy side. The fusion device was placed via the affected side and was a normal bullet type fusion device.

### Creatine kinase

Serum levels were measured using a spectroscopic method. Normal CK levels were considered to be 20–200 U/L.

Pain intensity measured by ‘worst pain in the past week’ on a Visual Analogue Scale (VAS; 0–10) [7, 8] and Oswestry Disability Index (ODI) [9]. Japanese Orthopaedic Association (JOA) [10] scores out of 29. < 10 points: poor; 10–15 points, moderate; 16–24 points, good; 25–29 points, excellent. Treatment improvement rate = [(post-treatment score − pre-treatment score) ÷ (out of 29 − pre-treatment score)] × 100%. ≥ 75%, excellent; 50–74%, good; 25–49%, moderate; 0–24%, poor. The improvement rate also corresponds to the commonly used criteria for determining efficacy: cure when the improvement rate is 100%, significant when the improvement rate is greater than 60%, effective when 25–60%, and ineffective when it is less than 25%.

Angular bisector measurement was used to measure superior intervertebral height. Viewer (2020.1.1) software measured the total area of bilateral multifidus muscles in the diseased interdisc segment versus the net area of multifidus muscles. Atrophy ratio of the patient's multifidus muscle = (preoperative area − postoperative area)/preoperative area.

### Statistics

The SPSS 17.0 software was used for statistical analysis. The *χ*^2^ test was used to compare the gender of the two groups. Age and the data of the preoperative, postoperative and follow-up observations were compared by two-tailed t-tests, and P < 0.05 was considered statistically significant.

## Results

In total, we included 46 single-level lumbar degenerative diseases at our hospital between September 2019 and December 2021. 22 in the study group and 24 in the ctrl group. There was no difference in age, gender, herniated segment of the lumbar spine, intervertebral fusion, and Cauda equina symptoms and decreased muscle strength between two groups (Table 1).

**Table 1:** Basic information of patients in two groups

Item	Ctrl group (*n* = 24)	Study group (*n* = 22)	*t*/*χ*^2^	*P*
Age (years)	51.04 ± 10.17	49.59 ± 9.610	0.496	0.622
Gender (male/female)	12/12	12/10	0.095	0.758
Segment (*n*)			0.382	0.536
L45	12	13		
L5S1	12	9		
Intervertebral fusion			0.1377	0.7106
Yes	21	20		
No	3	2		
Intervertebral fusion rate (%)	87.5	90.9		
Cauda equina symptoms and decreased muscle strength (n)	0	2	2.087	0.149

**Fig. 1:**
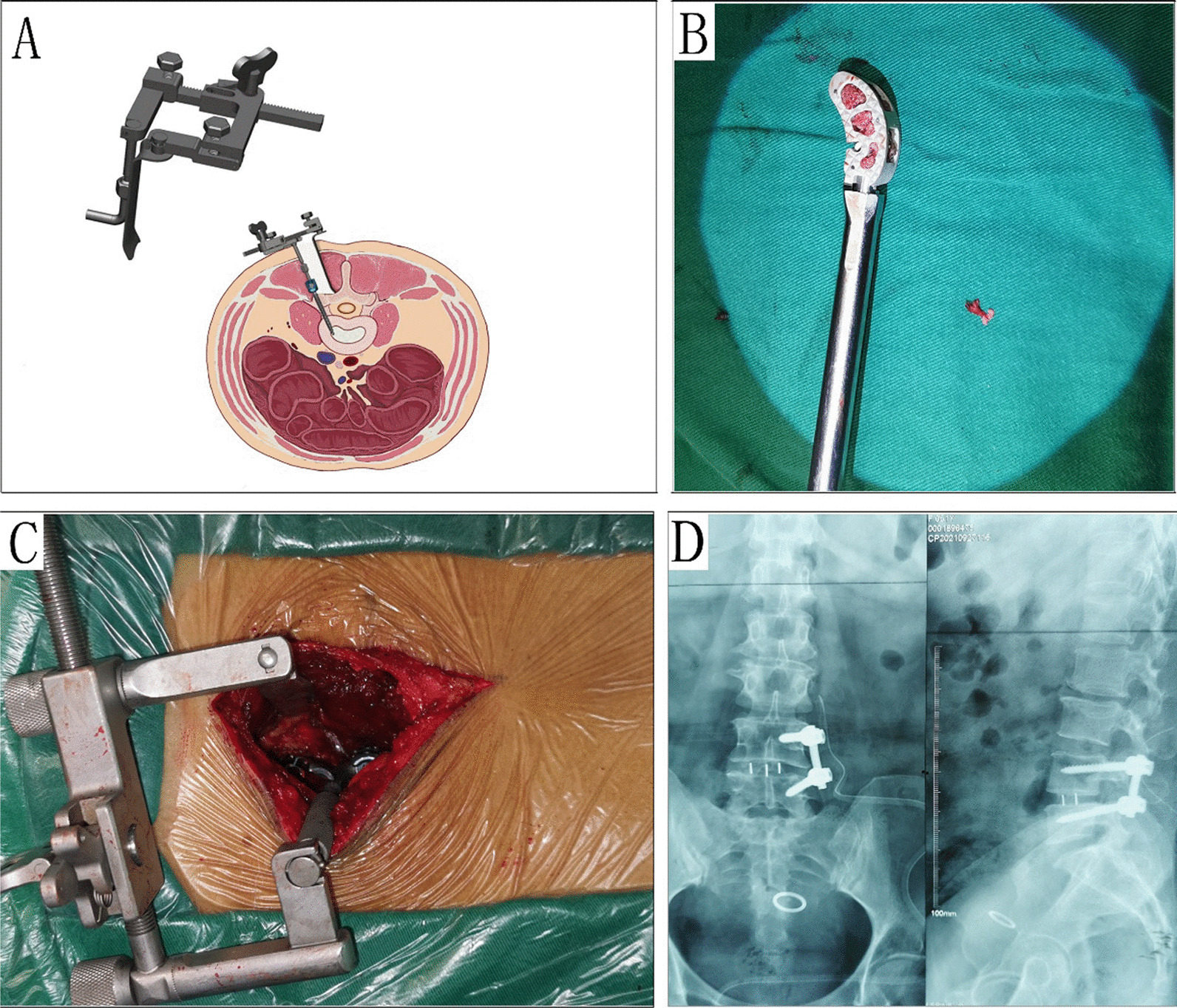
Illustrations of a new automatic retractor-assisted unilateral wiltse interval access TLIF procedure combined with unilateral nail bar fixation for single-segment lumbar degenerative disease. **A** Schematic diagram of the retraction device and the procedure. **B** Image of the renal peek fusion device. **C** Illustration of automatic retraction device in surgery. **D** CT image of unilateral screw fixation

In order to satisfactorily expose the intervertebral foramen and even the lateral aspect of the lumbar vertebrae through the Wiltse interval for the treatment of disc herniations, we have designed a new automatic retraction device (Fig. 1A, C). The new retraction device has been designed with an L-shaped support column at the fixed end, which is fixed to the pedicle screw at the caudal end of the segment with a nail cap. The support end is designed as an angle-adjustable retractor stop on the axial plane, which pushes the multifidus muscle towards the midline. Also, we use a kidney-like fusion cage to maintain spinal stability after unilateral pedicle screw fixation (Fig. 1B, D).

**Fig. 2:**
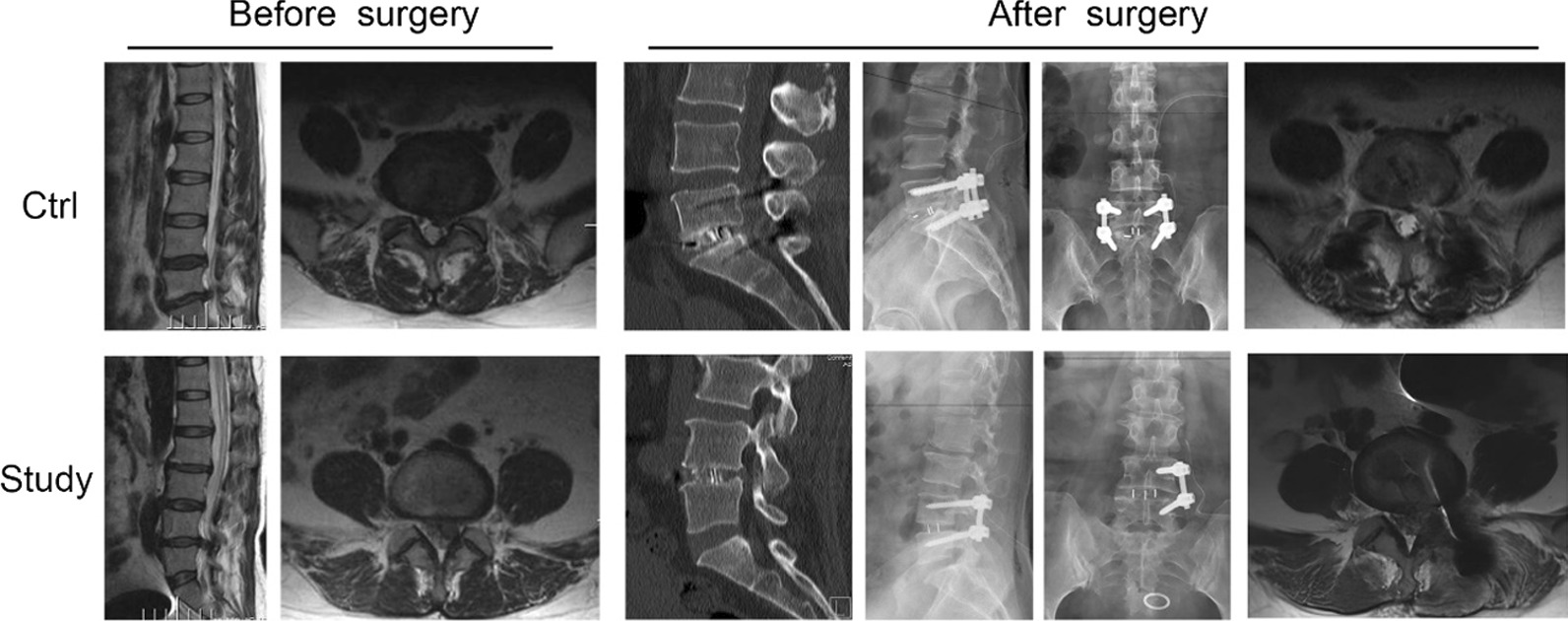
Images shown from left to right are MR sagittal; MR axial; CT sagittal; X-ray lateral; X-ray frontal and MR axial

We show the intervertebral imaging changes in the ctrl and study groups before and after surgery, with postoperative CT showing optimal intervertebral fusion in both the ctrl and study groups (Fig. 2).

**Fig. 3:**
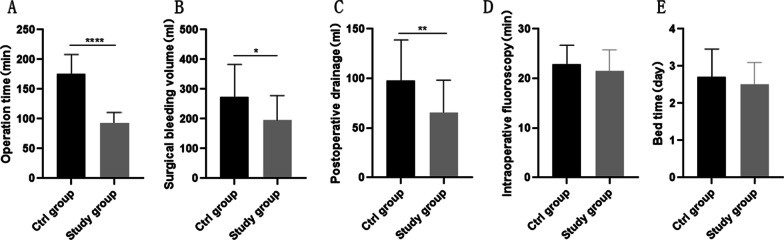
Differences in indicators between the two groups during surgery. **A** Operation time of two groups. **B** Surgical bleeding volume in two groups. **C** Postoperative drainage volume of two groups. **D** Intraoperative fluoroscopy time of two groups. **E** Postoperative bed rest time in both groups. *P < 0.05; **P < 0.01; ****P < 0.0001

For intraoperative fluoroscopy time and bed rest time, no differences were found between two groups. The operation time in study group was significantly shorter than ctrl group. Moreover, we also found that the study group had less surgical bleeding and postoperative drainage (Fig. 3A–E).

**Fig. 4:**
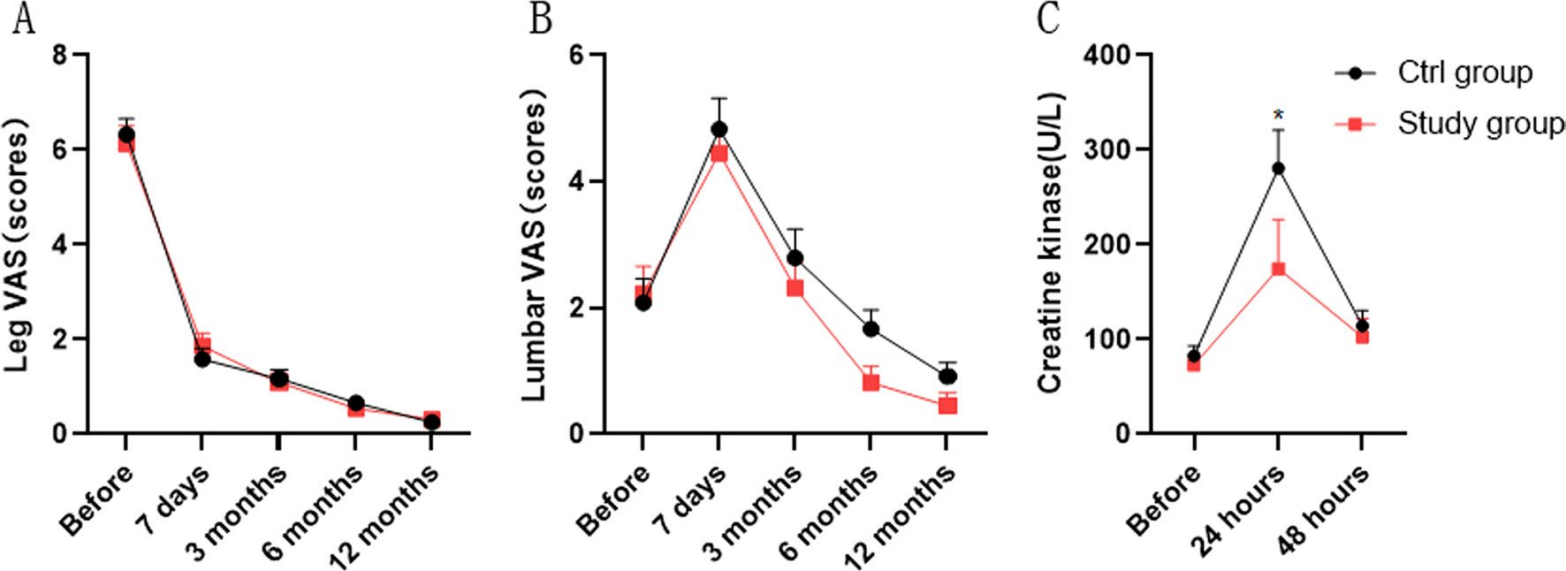
VAS and CK in two groups. **A** Preoperative and postoperative leg pain level in two groups. **B** Preoperative and postoperative lumbar pain level in two groups. **C** Changes in preoperative and postoperative creatine kinase in the two groups. *P < 0.05

To evaluative pain level of two groups, we recorded VAS scores before operation and during 1 year of post-operation. No differences were found for leg pain VAS and low back pain VAS in two groups (Fig. 4A, B). Creatine kinase is used to reflect muscle damage during surgery. Creatine kinase in study group was lower than ctrl group after operation for 24 h. For 48 h after operation, no differences were found in two groups (Fig. 4C).

**Fig. 5:**
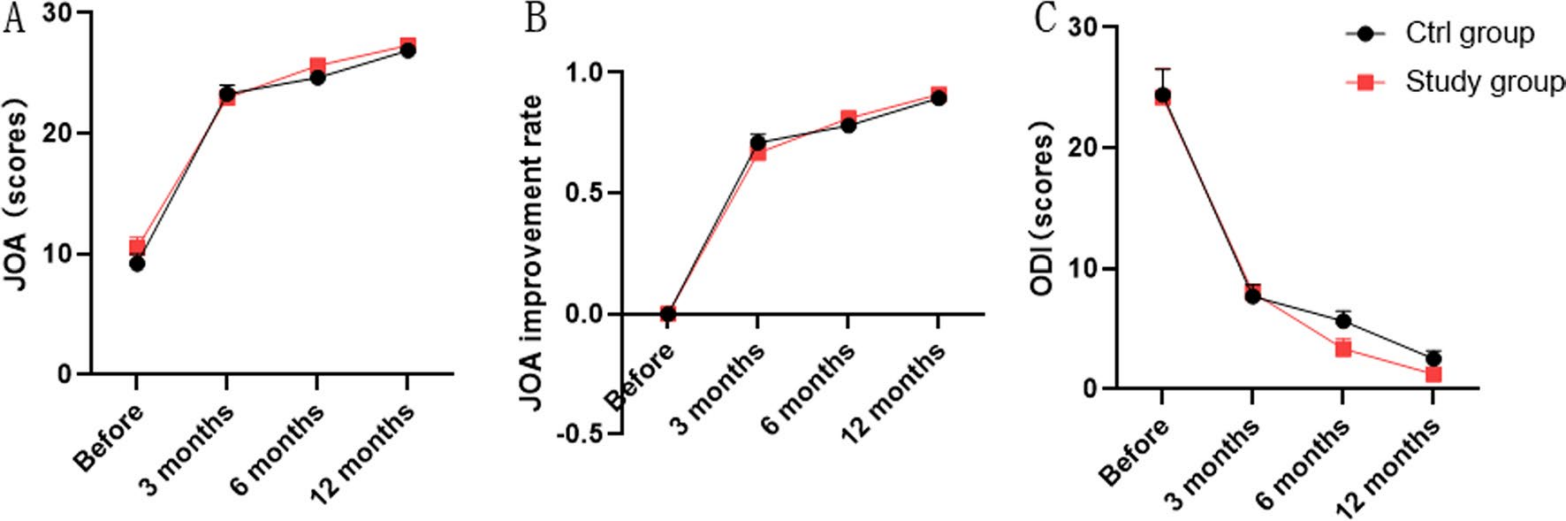
JOA and ODI in two groups. **A** JOA scores of surgical effectiveness in two groups. **B** Improvement rate of postoperative JOA in two groups. **C** Postoperative dysfunction index ODI in two groups

We determined the JOA and ODI scores of the two groups to evaluate the effect of surgery on lumbar spine function and found no significant difference between the two groups before surgery and during one year of follow-up (Fig. 5A–C).

**Fig. 6:**
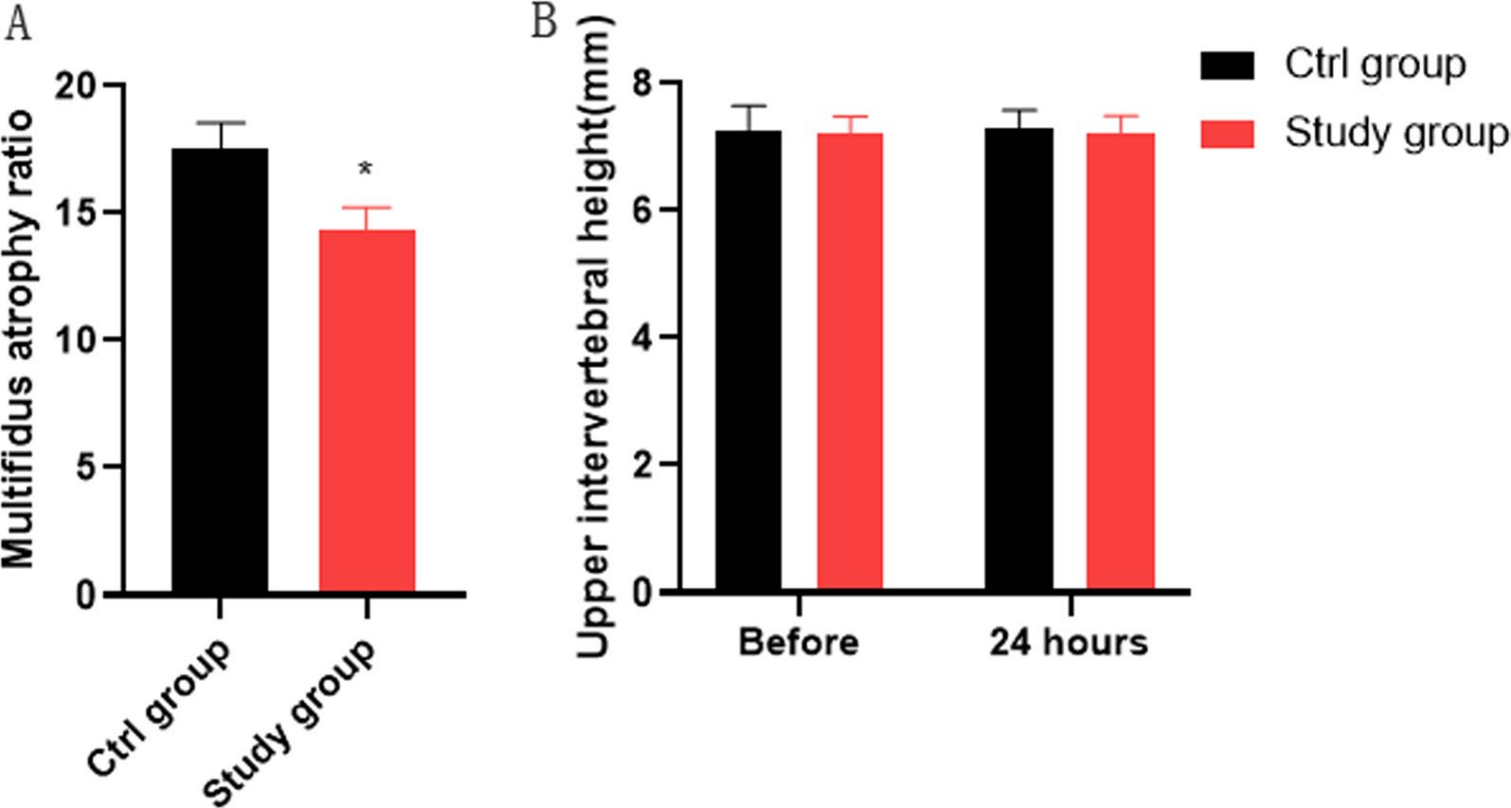
Multifidus atrophy ratio and upper intervertebral disc height in two groups. **A** Less muscle damage in the study group. **B** Surgery in both groups does not affect the Upper vertebral space height. *P < 0.05

We did not find a significant change in upper vertebral space height and modified Pfirrmann grading in the ctrl and study groups preoperatively and 1 year postoperatively (Table 2; Fig. 6B), which indicates surgery in both groups does not have a harmful effect on the adjacent segments of the lumbar spine. Moreover, the ratio of multifidus atrophy in patients was significantly lower in the study group than in the ctrl group, which implying a lower degree of damage to the multifidus muscle in the study group (Fig. 6A).

**Table 2:** Modified Pfirrmann grading in two groups

Item	Ctrl group (*n* = 24)	Study group (*n* = 22)	*t*/*χ*^2^	*P*
Preoperation modified Pfirrmann grading (*n*)			2.105	0.5509
Grade 2	7	6		
Grade 3	11	11		
Grade 4	4	5		
Grade 5	2	0		
Postoperation Modified Pfirrmann grading (*n*)			6.049	0.1955
Grade 2	7	6		
Grade 3	11	10		
Grade 4	2	6		
Grade 5	3	0		
Grade 6	1	0

## Discussion

The main muscles around the lumbar spine are divided into two groups: the anterior group includes the psoas major and psoas square, and the posterior group consists of the multifidus and sacrospinous muscles. In the postero-lateral region of the lumbar spine, three separate muscle gaps are formed between the multifidus, longissimus, iliopsoas muscle and lumbar square muscle. The longissimus has relatively small muscle fibers and forms a clear muscle gap with the medial multifidus muscle, which can be filled with a small amount of fatty tissue and can be easily separated bluntly without vascular or nerve penetration [11]. The Wiltse approach has been widely used in lumbar spine surgery. The current wiltse muscle gap approach to TLIF surgery is more damaging to the posterior spinal musculature and is not conducive to the preservation of low back muscle function [12]. The new retraction device in our study is designed with an L-shaped support post, which is fixed to the pedicle screw at the caudal end of the segment with no excessive pulling effect on the longest lateral muscle. The support end is designed as an angle-adjustable detent in the axial plane to "push" the multifidus muscle towards the midline. The retractor device is secured once a suitable position has been obtained. The area to be decompressed is clearly exposed. Direct visualization of the affected synovial joint, the vertebral plate and the base of the spinous process minimizes damage to the posterior spinal musculature during surgery, making it fully feasible for a single operator, especially in lower lumbar surgery. The multifidus muscle is tightly attached to both sides of the spinous process of the lumbar vertebrae, and the muscle bundle gradually thickens from the top downwards [13]. At the L1-L3 level, the gap is close to the midline and the small joints and transverse processes of the lumbar spine can be easily revealed, whereas at the L4-S1 level, the Wiltse gap deviates from the midline and the angle of inclination of the gap becomes greater [14]. It can be seen that the Wiltse gap can safely reveal different structures at different levels of the lumbar spine. The new automatic retractor we have designed makes intra-vertebral manipulation easy and convenient.

The advantages of unilateral pedicle screw fixation over bilateral pedicle screw fixation are: soft tissue damage is reduced by stripping only the paravertebral muscles on the affected side, preserving the structural integrity of the contralateral muscles and the posterior median ligament, reducing postoperative scarring of the paravertebral muscles and localized denervated atrophy, minimizing the occurrence of intractable postoperative low back pain [15]. For unilateral pedicle screw fixation, the spinal bony structures are reduced and the healthy side of the vertebral plate and articular processes are preserved, while maintaining the structural integrity of the spinal canal. The intraoperative bleeding, operating time and costs are significantly less than for bilateral pedicle screw fixation [16]. In our study, although we have cut off the inferior articular eminence, we retained the superior articular eminence as well, and we confirmed that the unilateral fixation group had significantly less operative time, intraoperative bleeding and postoperative drainage than the bilateral fixation group. H Xue et al. also found that a modified unilateral TLIF with only a unilateral screw had the advantages of less trauma, less bleeding and fewer complications, with a satisfactory clinical outcome rate better than conventional TLIF with bilateral pedicle screw fixation [17].

A three-dimensional finite element model was used to build the relevant surgical model, and biomechanical analysis revealed that single segment unilateral pedicle screw fixation without interbody fusion could not control the lateral flexion and rotation load well, and the pedicle screws were subjected to higher stresses; once a single fusion device was attached, the stability of the fused segment could be reestablished, and the stresses on the screws were significantly reduced [18]. After unilateral internal fixation with an intervertebral fusion, the stress on the pedicle screws was significantly reduced, although the peak stress was still higher than that of bilateral pedicle screw fixation, but the difference was minimal. This suggests that the intervertebral fusion device can significantly reduce the stress on the screw, thus reducing the incidence of screw fracture. Unilateral and bilateral internal fixation have similar resistance to flexion, extension, and compression, and unilateral pedicle screw fixation with a single fusion placement may provide sufficient stability as an option for internal fixation in the treatment of degenerative lumbar spine disease. Previous mechanical experiments using animal specimen modeling revealed that the mean stress intensity and axial stiffness of the lumbar spine after fusion with ipsilateral, contralateral, and bilateral pedicle screw fixation combined with fusion interbody fusion were significantly higher than those of simulated injury specimens and normal specimens, and that fusion implantation with additional unilateral and bilateral pedicle screw system internal fixation could achieve stabilization of the lumbar spine. The biological stability of bilateral specimens in the directions of forward flexion, left bending, and left and right rotation did not differ between the unilateral model and the bilateral model [19]. A large number of in vitro tests have confirmed to some extent the feasibility and stability of unilateral pedicle screw fixation combined with fusion of the intervertebral fusion. Unilateral decompression is difficult to perform intra-vertebral interspace double fusion placement. The common peek bullet type interbody fusion is not accurate for maintaining stability of the anterior and middle spinal columns, especially when the patient is in a twisted and lateral position, due to the limited angular tilt during transmural placement and the small cross-section and bone contact area of the fusion [20]. Moreover, the unilateral nail bar combined with the bullet-type fusion cage suffers from stress imbalance, does not provide sufficient stability due to its asymmetric fixation, and is less resistant to rotation with unilateral internal fixation [21]. To overcome this problem, we adopted a kidney-like fusion cage with more uniform stress distribution. The kidney-like fusion is larger and has a larger contact area with the bony endplate, and the extra-large bone graft window allows for greater bone filling. When the kidney-like fusion is placed into the intervertebral space, the forces on the left and right sides of the anterior mid column become equalized. The implantation of a renal fusion after simultaneous gap sparing and the restoration of tension in the anterior and posterior longitudinal ligaments both reduce the impact on the healthy synovial joint during torsion and lateral bending, and the preservation of synovial mobility on the healthy side reduces the occurrence of accelerated degeneration in the adjacent segment. Overall, the use of unilateral screw fixation with a kidney-like fusion device can maintain three-column stability, no less than bilateral fixation. It also preserves the partial mobility of the operated segment and reduces the occurrence of adjacent segment disease.

The limited resection of bony structures in the posterior column and the small amount of autologous bone obtained by the interosseous approach TLIF procedure do not meet the need for intervertebral fusion bone grafting [22]. Currently, alternative grafts for clinical use include allograft bone, synthetic bone, and bone morphogenetic protein (BMP). Allogeneic bone is mainly derived from cadaveric tissue, including freeze-dried bone, fresh frozen bone, cancellous bone strip bone, and decalcified bone matrix (DBM), which are inexpensive and easily available [23]. BMP is a specific bone growth factor, which can form new bone in ectopic or normal location through chondrogenic bone and intramembranous bone, and has strong bone repair ability [24]. However, BMP can only act locally and is easily diffused and degraded in vivo, and easily loses its activity. In our study, the rate of intervertebral fusion was high in two group. Since the study group using both allogeneic bone and BMP to replace autologous bone in the intervertebral space, fusion rate appear to be higher, which indicating that good fusion results can be obtained with kidney-like fusion cage.

Pfirrmann et al. developed a system for classifying disc degeneration based on MR signal intensity, disc structure, distinction between the nucleus pulposus and nucleus pulposus, and disc height, which with 5 grades and has been accepted and used in clinical applications [25]. However, the Pfirrmann Grading System was previously found to be ambiguous in classifying intervertebral discs, so the modified Pfirrmann grading system, which has 8 grades, is more commonly used in clinical practice to better distinguish the degree of disc degeneration [26]. Our study found no difference in Modified Pfirrmann Grading of the upper intervertebral disc between the study and control groups, either preoperatively or postoperatively. The height of the intervertebral space of the upper intervertebral disc can be used to reflect the degeneration of the lumbar spine [27]. We found no significant difference in the intervertebral space height of the upper intervertebral disc between the two groups, either preoperatively or postoperatively. It is possible that both types of surgery are effective in treating herniated discs, with no harmful impact on the nearby segments.

There are 3 isozymes of creatine kinase, mainly found in skeletal muscle, brain and cardiac muscle. Since plasma levels of skeletal muscle isoenzymes account for more than 96% of the total, creatine kinase values can be used to reflect skeletal muscle damage in surgery that does not affect the brain or myocardium. Creatine kinase is an enzyme that catalyzes the reaction of creatine and adenosine triphosphate (ATP) with phosphocreatine and adenosine diphosphate (ADP). Creatine kinase concentrations have been used to study skeletal muscle damage caused by lumbar spine surgery [28]. The elevation of creatine kinase isoenzymes in skeletal muscle is mainly related to the process of muscle damage and is generally highest on the first postoperative day, returning to normal by 1 week after surgery [29]. In our study, creatine kinase levels increased significantly at 24 h after surgery and decreased significantly at 48 h. Interestingly, Creatine kinase levels were significantly lower in the study group than in the ctrl group at 24 h postoperatively, suggesting that the unilateral wiltse muscle gap access TLIF procedure combined with the treatment procedure was less damaging to the muscle.

Some deficiencies exist in our study, first, the sample size of patients was single-centered and insufficient in number. Second, in addition to the difference between unilateral and bilateral fixation treatment, our study differs from the ctrl group used a kidney-like fusion cage in the unilateral treatment group, and it is not well distinguishable whether unilateral fixation or kidney-like fusion cage or their combined effect has a greater impact on the outcome, and more subsequent studies are needed to confirm this.

## Conclusion

We designed a novel automatic retraction device to assist unilateral wiltse muscle gap access TLIF surgical fixation for single-level lumbar degenerative diseases with a kidney-like fusion cage to prevent cage subsidence. The application reduced blood loss during surgery and operative time, and showed better protection of back muscle function during lumbar degenerative diseases treatment, with great potential for clinical application.

## Data Availability

The datasets generated and analyzed during the current study are available from the corresponding author on reasonable request.
